# Evaluation of the Fusion Rate and Safety of *Escherichia coli*-Derived rhBMP-2 in Transforaminal Lumbar Interbody Fusion for Patients with Degenerative Lumbar Disease: A Prospective, Multicenter, Single-Arm Trial

**DOI:** 10.3390/jcm13061733

**Published:** 2024-03-17

**Authors:** Ji-Won Kwon, Jae Hwan Cho, Jong Beom Lee, Jae Hong Kim

**Affiliations:** 1 Department of Orthopedic Surgery, Yonsei University College of Medicine, Seoul 03722, Republic of Korea; kwonjjanng@yuhs.ac (J.-W.K.); laehong892@yuhs.ac (J.H.K.); 2 Department of Orthopedic Surgery, Asan Medical Center, University of Ulsan College of Medicine, Seoul 05505, Republic of Korea; 3 Department of Neurosurgery, Chungbuk National University Hospital, Cheongju 28644, Republic of Korea; shock33-1@hanmail.net

**Keywords:** fusion rate, transforaminal lumbar interbody fusion, lumbar, rhBMP-2

## Abstract

**Background:** Few studies have documented the viability of *E. coli*-derived recombinant human bone morphogenetic protein-2 (rhBMP-2) in transforaminal lumbar interbody fusion (TLIF). This study aimed to assess the safety and fusion rate of rhBMP-2 in TLIF. **Methods:** The study was conducted as a prospective, multicenter, single-arm trial, and 30 patients needing one- or two-level TLIF were enrolled. Fusion rate was assessed using the 12-month interbody fusion rate on CT. Postoperative problems, including seroma, radiculitis, and ectopic bone formation, which have been documented as risks associated with rhBMP-2 in prior studies, were recorded. **Results:** The study demonstrated fusion outcomes in all instances at 52 and 104 weeks post-surgery. Significant improvements were observed in clinical outcomes, with ODI, SF-36, and VAS scores, all achieving statistical significance (*p* < 0.0001). No perioperative adverse events requiring reoperation were reported, and there were no incidences of seroma, radiculitis, cage migration, grafted bone extrusion, postoperative neurologic deficit, or deep wound infection. **Conclusions:** The study demonstrates the high safety and efficacy in inducing bone fusion of *E. coli*-derived rhBMP-2 in TLIF, with a notable absence of adverse postoperative complications. Trial registration: This study protocol was registered at Korea Clinical Research Information Service (number identifier: KCT0004738) on July 2020.

## 1. Introduction

Degenerative lumbar disorders, such as lumbar spondylosis, spinal stenosis, and spondylolisthesis, are a major source of chronic back pain and disability, affecting millions of people globally. Various studies show surgical intervention is often unnecessary and leads to short- or long-term complications [1]. However, in cases where conservative treatments fail to be effective, surgical intervention becomes an inevitable option due to the progressive nature of these conditions.

Several surgical intervention techniques for degenerative disorders have already been documented [2]. Transforaminal lumbar interbody fusion (TLIF) has become a commonly used surgical technique for these conditions, providing relief by relieving pressure and stabilizing the afflicted parts of the spine. The achievement of a stable spinal fusion is crucial for the success of TLIF [3,4,5], and this outcome is impacted by several factors such as patient characteristics, surgical technique, and the choice of fusion materials. Traditionally, autografts have been considered the most reliable method for spinal fusion because of their ability to promote bone formation, stimulate bone growth, and provide a scaffold for new bone tissue [6,7]. Nevertheless, the investigation of substitute graft materials has been prompted by constraints such as donor site morbidity and restricted availability. Of these materials, bone morphogenetic proteins (BMPs) have attracted considerable interest. Recombinant human bone morphogenetic protein-2 (rhBMP-2) has been thoroughly researched and utilized because of its powerful ability to stimulate bone formation. BMPs are a collection of growth factors that are recognized for their capacity to stimulate the differentiation of mesenchymal stem cells into osteoblasts, which are responsible for bone formation [8,9]. Utilizing rhBMP-2 in spinal fusion has been documented to augment fusion rates, diminish the necessity for supplementary operations, and boost clinical results. Nevertheless, the origin of rhBMP-2 is a critical determinant that can impact its effectiveness and safety. Historically obtained from mammalian cells, recent progress has made it possible to produce rhBMP-2 from *Escherichia coli*, which could provide a more reliable and safer product [10,11]. Further research is needed to thoroughly examine the use of *E. coli*-derived rhBMP-2 in clinical settings, namely in TLIF operations for degenerative lumbar illnesses, despite its demonstrated osteoinductive potential in preclinical models. This study aims to evaluate the fusion rate and safety profile of *E. coli*-derived rhBMP-2 (NOVOSIS^®^, CGbio, Inc., Seoul, Republic of Korea) in TLIF among patients with degenerative lumbar diseases. By comparing these outcomes with existing literature on traditional BMP sources and other fusion materials, we aim to provide a comprehensive understanding of its role in modern spinal surgery.

## 2. Materials and Methods

### 2.1. Study Design

From April 2021 to December 2023, a total of 30 patients requiring posterior instrumentation and TLIF for degenerative conditions between L2 and S1 were enrolled. This study had the participation of three orthopedic spine surgeons who had over 5 years of experience after completing their fellowships. These surgeons were affiliated with three separate tertiary care institutions and actively recruited patients for the study. Patients aged 18 to 75 years, diagnosed with lumbar spinal stenosis, herniated disc, or spondylolisthesis, and needing single or two-segment TLIF between L2 and S1 were considered. Exclusion criteria included severe metabolic bone diseases (e.g., Paget’s disease), severe osteoporosis (BMD, bone mineral density T-score < −3.0), ongoing systemic or acute infections, a history of drug or alcohol abuse within two years, a history of malignancy (unless in remission for over 5 years), pregnancy, and inability to discontinue bone metabolism-affecting medications. Participants were required to visit the outpatient clinic after surgery six times; post-operative days 4, 12, 26, 39, 52, and 104 weeks. At each visit, clinical assessments, imaging studies including plain X-ray and computed tomography (CT), and rhBMP-2 related safety evaluations were conducted. All subjects gave their informed consent for inclusion before they participated in the study. The study was conducted in accordance with the Declaration of Helsinki, and the protocol was approved by the Ethics Committee of Asan Medical Center (IRB number: 20201092, Ethics Approval Date: 10 July 2020). In addition, this study was registered at Korea Clinical Research Information Service (https://cris.nih.go.kr, [accessed on 10 July 2020]; number identifier: KCT0004738) in July 2020.

### 2.2. Surgical Procedure

**Figure 1:**
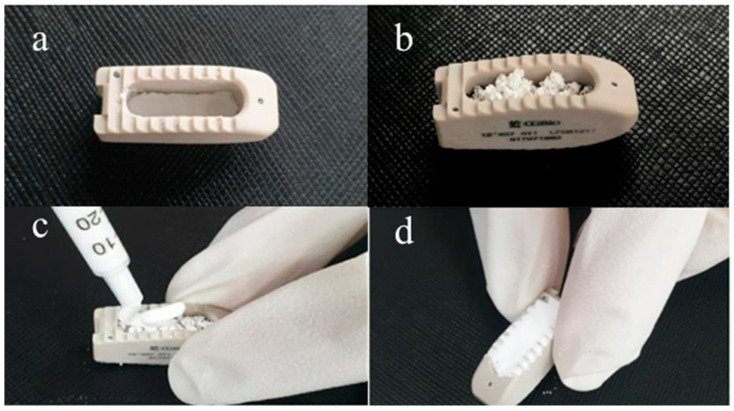
Description of the process of soaking rhBMP-2 in a TLIF cage. (**a**) Place Excelos Inject on the bottom of the window within the cage. (**b**) Fill the cage with HA-containing bone graft material soaked with rhBMP-2 at a dose of 0.5 mg to 1.0 mg per fusion segment. (**c**) To fix the graft material in the cage, apply the remaining Excelos Inject to the surface covered with the bone graft material. (**d**) Mold to fit the shape of the cage to prevent leakage when inserted into the interbody space. Abbreviations: rhBMP-2—recombinant human bone morphogenetic protein-2; HA—hydroxyapatite.

All three surgeons who were present utilized comparable procedures when executing the TLIF procedure [12]. Following the insertion of pedicle screws, a surgical procedure called inferior facetectomy is carried out using osteotomes via the same side’s pars. The interspinous ligament located between the surgical levels is removed, and a laminar spreader is employed to facilitate the separation of the disc space in anticipation of the discectomy. Ipsilateral pedicle screw distractors are employed for secondary distraction. A high-speed burr or osteotome is utilized to remove the superior facet until it is level with the distal pedicle, therefore revealing the transforaminal corridor. After removing the bone, the ligamentum flavum on the same side is surgically removed using kerrisons. This exposes the thecal sac and the nerve root that passes through it. The bones surrounding the caudal pedicle are removed using kerrisons in order to obtain a clear view of the neural processes. The disc space is located, and bipolar cautery is employed to cauterize epidural veins. A right-angled neural protector is employed to retract the thecal sac towards the center and safeguard the departing nerve root. A discectomy is carried out using disc space shavers, currettes, and rasps. Trials are conducted to determine the suitable dimensions of a cage, specifically the height and width, in order to place the broadest possible TLIF structural polyetheretherketone cage (Capstone; Medtronic, Memphis, TN) that can achieve the maximum surface contact area. The dosage of recombinant human bone morphogenetic protein-2 (rhBMP-2) was determined to be measured and administered in a range of 0.5 to 1.0 milligrams per fusion level. The procedure for encapsulating rhBMP-2 within the TLIF cage is as follows. Administer Excelos Inject (CGbio, Inc., Seoul, Republic of Korea), a biocompatible ceramic composed of β-Tricalcium phosphate and poloxamer 407, to the lower part of the cage. Then, fill the cage with hydroxyapatite (HA) bone graft material that has been saturated with the matching rhBMP-2. Furthermore, during the process of cage implantation, an Excelos injection is administered to prevent any potential leakage of the bone graft material from the window within the cage (Figure 1).

### 2.3. Radiologic Outcomes

**Figure 2:**
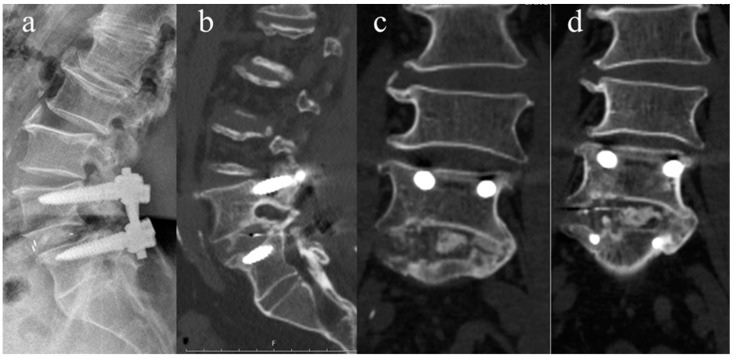
Representative plain X-ray and CT slices of a 72-year-old man who underwent 1 level TLIF. (**a**) Plain X-ray at 52 weeks after surgery, Grade I according to Bridwell classification (fused with remodeling and trabeculae present); (**b**–**d**) Grade II, which refers to partial fusion where the allografts cortex joins with the endplate in CT sagittal and coronal view. Abbreviations: CT—computed tomography.

The primary efficacy endpoint was the fusion rate at 52 weeks post-surgery assessed by CT scans. Fusion was defined as the presence of continuous bridging bone across the treated segment without any gaps. Secondary efficacy measures included fusion rates at various intervals (26, 39, and 104 weeks) assessed by both X-ray and CT. Fusion rates were analyzed for each operated segment. The patients were graded by the two independent orthopedic spine surgeons, who were each blinded to the other’s response and clinical outcomes into one of four grades of fusion according to CT appearance. When there is Grade I fusion (complete fusion), the allograft’s cortical union at the cranial and caudal ends of the bone is present, as well as continuity of the trabecular pattern between the autograft and the neighboring cranial and caudal vertebral bodies in the medullary canal of the allograft. There is some or no trabecular continuity between the medullary autograft and the neighboring vertebral body bone at one or both ends in Grade II (partial fusion). This means that the allograft’s cortex joins with the endplates on both ends. Grade III (unipolar pseudarthrosis) means that either the cranial or caudal cortical non-union of the allograft is present, along with a central trabecular discontinuity. Grade IV (bipolar pseudarthrosis) means that both the superior and inferior cortical non-union are present, along with a total lack of central trabecular continuity [13]. Radiographic fusion using plain X-ray was assessed by anterior fusion grades described by Bridwell et al. [14,15]. Grade I, fused with remodeling and trabeculae present; Grade II, graft intact but not fully remodeled and incorporated, with no lucencies above or below; Grade III, graft intact but with a definite lucency at the top or bottom of the graft; and Grade IV, definitely not fused, with resorption of bone graft and collapse. Both Grades I and II were considered signs of radiographic fusion (Figure 2). The intraobserver reliability was evaluated by presenting the instances to the same examiners, albeit in a different sequence, after a period of 6 weeks. The estimation of inter- and intraobserver reliability was conducted by computing the kappa coefficient. The agreement between the examiners was assessed using the Landis and Koch categorization.

### 2.4. Clinical Outcomes

Clinical outcome measures included the Oswestry Disability Index (ODI) [16,17], Short Form-36 (SF-36) health survey scores [18], and Visual Analogue Scale (VAS) for back and leg pain [19]. The ODI was estimated using the Oswestry Low Back Pain Disability Questionnaire. Scores on the ODI range from 0 to 100 points, with lower scores representing less pain and better function. Back and leg pain scores were assessed with a 10-point numeric rating scale, where 0 was defined as no pain and 10 as very severe pain. The SF-36 comprises eight scaled scores, which are calculated by assigning weights to the questions within each section and summing them. Each scale is linearly converted to a 0–100 scale, assuming that each question holds equal significance. A lower score indicates a higher degree of disability. Adverse postoperative complications associated with rhBMP-2 were collected through follow-up up to 2 years after surgery. Adverse events that were not specifically related to spine surgery and did not affect recovery (for example, urinary tract infection, ileus, anemia) were excluded.

### 2.5. Statistical Analysis

All analyses were conducted using SPSS Statistics, version 25 (IBM Corp., Chicago, IL, USA). The study employed a modified intention-to-treat approach, using a Full Analysis Set (FAS) for the primary analysis. The Per Protocol (PP) set was also analyzed. Continuous variables were summarized using descriptive statistics (mean, standard deviation, median, minimum, maximum), and categorical variables were presented in frequencies and percentages. Statistical significance was set at a *p*-value of <0.05, and 95% confidence intervals were calculated.

## 3. Results

### 3.1. Demographic Characteristics and Operative Data

After undergoing TLIF surgery with rhBMP-2, a cohort of 30 patients participated in a prospective study. The age distribution was 65.4 ± 7.7 years, with a gender ratio of 20 men to 10 women. Lumbar spinal stenosis constituted the most prevalent preoperative diagnosis at 70%, with a total fusion segment level of 38 and L4–5 accounting for 50% of the cases. Hypertension emerged as the prevailing coexisting medical condition (Table 1).

### 3.2. Radiographic Fusion Using CT and Plain Radiography

A progressive increase in spinal fusion rates over time was noted, as assessed by both CT and plain radiography. Initially, at 26 weeks post-surgery, the fusion rate was 83.87%, increasing slightly to 80.77% by 39 weeks. Remarkably, by the 52-week mark, and continuing at 104 weeks, a 100% fusion rate was achieved, demonstrating successful fusion across all evaluated segments. Using Landis and Koch’s measurement, the mean kappa coefficient value for interobserver reliability was determined to be 0.92, and for intraobserver reliability after 6 weeks it was 0.88. These values indicate that there is nearly perfect agreement between observers and time intervals (Table 2).

### 3.3. Clinical Outcomes

Clinical results improved significantly across numerous parameters. The ODI showed a significant decrease in impairment levels for back pain patients, from 62.89 ± 19.24 to 24.30 ± 18.99 after 104 weeks. The SF-36 Health Survey revealed a considerable improvement in quality of life, rising from 47.56 ± 25.42 to 63.36 ± 22.13 after 104 weeks. VAS pain also decreased significantly. VAS scores for back pain decreased from 53.10 ± 27.09 to 19.45 ± 16.18 at 104 weeks, demonstrating satisfactory post-surgery pain management. At 104 weeks, leg pain VAS scores significantly decreased from 59.20 ± 33.12 to 20.95 ± 21.24 (Table 3).

### 3.4. Adverse Events Related to Surgeries

In the study assessing the safety of rhBMP-2, the evaluation of adverse postoperative complications revealed encouraging results among the enrolled patients. Notably, there were no reoperations required due to perioperative adverse events, underscoring the procedural safety. Specifically, during the perioperative period, which includes up to 12 weeks post-surgery, there were zero incidences of seroma, radiculitis, cage migration, grafted bone extrusion, postoperative neurologic deficit, or deep wound infection among the 30 patients. Furthermore, in the postoperative period beyond 12 weeks, the study reported no cases of pseudoarthrosis, metal failure and/or screw breakage, or ectopic bone formation.

## 4. Discussion

The use of *Escherichia coli*-derived rhBMP-2 in spinal fusion procedures, namely in TLIF, represents notable progress in the realm of orthopedic surgery. The results of our investigation showed significant fusion rates and a positive safety profile of NOVOSIS in TLIF procedures. These findings align with previous research that highlights the potential of *E. coli*-derived BMP-2. rhBMP-2 has been proven to provide fusion rates that are either equivalent to or higher than those achieved with autologous iliac bone grafts in lumbar fusion procedures. BMP-2 promotes the development of osteoblasts and augments bone production, which is essential for the effective fusion of the spine [20]. The fusion rates observed in our study at the 52-week mark provide additional evidence of the osteoinductive capabilities of BMP-2. These findings are consistent with a previous study by Parker et al. [21], which also shown improved spinal fusion rates with the use of BMP-2. This is consistent with previous research, which emphasizes the effectiveness of rhBMP-2 in facilitating successful spinal fusions. *E. coli*-derived BMP-2 has various advantages in comparison to mammalian cell-derived BMP-2. The production technique of *E. coli*-derived BMP-2 is primarily more cost-effective and scalable, making it a more accessible choice for widespread clinical application. Furthermore, it has been proposed that BMP-2 produced from *E. coli* may demonstrate reduced immunogenic responses, a crucial factor to consider in its therapeutic uses [22]. Furthermore, a crucial factor to take into account when utilizing rhBMP-2 was the dosage. The study attempted to establish a safe threshold while preserving efficacy by utilizing a lower dose (0.5–1 mg per level of fusion) of *E. coli*-derived rhBMP-2. This decision is informed by past studies that did not find a significant correlation between increased rhBMP-2 dosage and improved fusion rates, thereby suggesting that smaller doses may be equally effective [23]. Hence, further investigation is required to determine the dosage of rhBMP-2 that can expedite the process of fusion. Further research related to this is currently in progress. It is expected that the results of the study conducted as a multicenter, randomized controlled trial will verify the efficacy of *E. coli*-derived BMP-2 [24].

The selection of a carrier for rhBMP-2 is an additional critical factor. The study employs HA as a carrier due to its osteoconductive capabilities and strong affinity for rhBMP-2. The utilization of hydroxyapatite (HA) as a medium for *E. coli*-derived rhBMP-2 in TLIF surgery is highly intriguing because of its well-established benefits in bone grafting procedures [25]. HA, a naturally occurring mineral composed of calcium apatite, possesses characteristics that render it very suitable for this particular function. To begin with, the biocompatibility of HA is a significant advantage. Due to its osteoconductive nature, it facilitates the formation of new bone by enabling bone cells to multiply and move into the scaffold. This characteristic is crucial for the effective integration of bone grafts. In spinal fusion procedures, the development of a robust bone bridge is of the utmost importance for ensuring the stability of the fused segments [26,27]. Moreover, the chemical and structural resemblance of HA to real bone tissue renders it a superb substance for bone grafting. The composition of this material closely resembles that of human bone, reducing the likelihood of immunological rejection and promoting the integration of the graft with the recipient’s bone. The permeable characteristics of HA also have a crucial impact. The interconnected pore network of the bone graft allows for the formation of blood vessels and the exchange of nutrients, which are crucial for the survival of bone cells and the overall effectiveness of the graft [28]. The property of HA is advantageous for the inclusion of growth factors such as rhBMP-2, since it enables a continuous release of these proteins, hence augmenting their ability to stimulate bone formation. Furthermore, HA is renowned for its long-lasting and steadfast nature, ensuring that it retains its form over an extended period. This characteristic allows for uninterrupted assistance during the crucial stages of bone healing and restructuring. Ensuring material integrity is crucial in spinal procedures, since any degradation could result in instability or fusion failure.

The utilization of *E. coli*-derived rhBMP-2 has economic benefits. After analyzing the latest research and our study’s findings, it is clear that *E. coli*-derived rhBMP-2 offers substantial economic benefits compared to Chinese hamster ovary (CHO) cell-derived rhBMP-2, especially for TLIF procedures [29]. The production costs and yield are crucial factors that distinguish between rhBMP-2 derived from *E. coli* and rhBMP-2 derived from CHO cells. The use of rhBMP-2 generated from *E. coli* is more cost-effective since it has reduced production expenses and the capacity to generate bigger amounts [30]. Furthermore, research has shown that the ability of *E. coli*-derived rhBMP-2 to stimulate bone growth is similar to that of CHO-derived rhBMP-2. Due to its comparable effectiveness and lower cost, *E. coli*-derived rhBMP-2 is a more feasible choice for extensive clinical application in spinal fusion procedures. The economic advantages of rhBMP-2 generated from *E. coli* surpass the immediate expenses associated with the product itself. Healthcare providers can decrease the overall expenses related to spinal fusion surgery by providing a bone graft option that is both cost-effective and effective in its purpose. This can be especially advantageous in healthcare environments, where the allocation of resources and cost-effectiveness are crucial factors. However, the initial costs of rhBMP-2 itself can be high, which is a significant consideration for healthcare providers and patients. Future research should focus on a comprehensive economic analysis comparing the cost of rhBMP-2-based treatments with traditional methods, considering both the short-term and long-term economic implications. Studies could also explore the cost-effectiveness of different dosages and formulations of rhBMP-2, as well as its use in various clinical settings.

In addition to its efficacy, the use of rhBMP-2 is advantageous due to its paramount focus on safety in the application of BMP-2. While rhBMP-2 is effective in promoting bone growth and healing, concerns have been raised about potential adverse effects, particularly at higher doses. The occurrence of unfavorable incidents in our research was negligible, and we did not find any noteworthy issues associated with the off-label utilization of rhBMP-2. Given the previous concerns about BMP-2, such as inflammation, radiculitis, seroma, ectopic bone growth, and probable carcinogenicity, this finding is of the utmost importance [31]. This study did not examine the correlation between the dosage of rhBMP-2 and the occurrence of postoperative problems in the TLIF operation. The study utilized a smaller dosage range of around 0.5 to 1 mg per fusion level, thereby preventing the identification of any association between the dosage and postoperative problems. Notably, around 40–50% of TLIFs performed in the United States utilized rhBMP-2 [23]. However, there have been limited studies examining the precise clinical results associated with different dosages of rhBMP-2 in TLIFs, except for the presence of radiculitis [32]. Notwithstanding these concerns, a study encompassing a substantial group of 642 patients over a span of seven years discovered no correlation between extensive exposure to high-dose rhBMP-2 and specific severe consequences such as cancer [33,34]. This discovery indicates that although certain difficulties are linked to increased dosages of rhBMP-2, not all serious negative consequences are directly proportional to the dosage. The safety profile shown in our investigation indicates that *E. coli*-derived BMP-2 could potentially reduce some of these hazards. However, additional long-term trials are necessary to validate these findings. Future research is crucial and should aim to further elucidate the mechanisms behind these adverse effects, explore the long-term outcomes of rhBMP-2 use, and determine the optimal dosages that balance efficacy and safety. Additionally, the development of alternative formulations or delivery systems for rhBMP-2 could help in mitigating these risks. Investigations into patient-specific factors that may influence the safety profile of rhBMP-2 are also important, as personalized approaches to its use could improve overall safety and efficacy.

However, it is important to acknowledge the limitations of this study. The single-arm design and the absence of a control group using a different type of BMP-2 or another graft material limit the extent to which these results can be generalized. For future research directions or possible applications of *E. coli*-derived rhBMP-2, it would be valuable to explore its efficacy in a broader range of spinal conditions and to compare its outcomes with other bone graft alternatives in randomized controlled trials. Investigating the optimal dosage and formulation, especially in conjunction with different carriers, can further refine its use. Long-term studies assessing the sustainability of fusion and any late-onset complications will also be crucial. Additionally, researching the cost-effectiveness of *E. coli*-derived rhBMP-2 in various healthcare settings could significantly impact its adoption in clinical practice. These areas of research will help to establish a more comprehensive understanding of the role of *E. coli*-derived rhBMP-2 in spinal surgery and orthopedic treatments.

## 5. Conclusions

In conclusion, our study adds to the growing body of evidence supporting the use of *E. coli*-derived BMP-2 in spinal fusion surgeries. The results underscore its efficacy in inducing bone fusion and safety, making it a promising alternative to traditional bone graft materials. Ongoing research and comparative studies will be crucial in cementing its role in orthopedic surgery.

## Figures and Tables

**Table 1:** Demographic characteristics and surgical data of patients. Abbreviations: BMI—body mass index; COPD—chronic obstructive pulmonary disease. Values are expressed as mean ± SD or percentages.

	Enrolled Patients (n = 30)
Age (years)	65.4 ± 7.7
Sex (Female/Male)	20/10
BMI (kg/m^2^)	26.1 ± 2.9
Current smoking status (Yes/No)	3/27
Preoperative diagnosis	
Lumbar spinal stenosis	21 (70.0%)
Recurrent lumbar disc herniation	1 (3.3%)
Isthmic or degenerative spondylolisthesis	8 (26.7%)
Number of treated segment	38
Treated spine level	
L2–L3	1 (2.6%)
L3–L4	7 (18.4%)
L4–L5	19 (50.0%)
L5–S1	11 (28.9%)
Coexisting medical conditions	
Hypertension	9 (30.3%)
Cerebrovascular disease	0 (0.0%)
Diabetes mellitus	2 (6.7%)
COPD or pneumonia	1 (3.3%)
Congestive cardiac failure	0 (0.0%)
Peripheral vascular disease	0 (0.0%)
Any solid tumor	0 (0.0%)

**Table 2:** Fusion rate using plain radiography and computed tomography by segments.

	26 w (n = 31)	39 w (n = 26)	52 w (n = 25)	104 w (n = 22)
Computed tomography				
Fusion, n (%)	26 (83.87)	21 (80.77)	25 (100.00)	22 (100.00)
Non-fusion, n (%)	5 (16.13)	5 (19.23)	0 (0.00)	0 (0.00)
Plain radiography				
Fusion, n (%)	25 (78.13)	22 (75.86)	29 (100.00)	22 (100.00)
Non-fusion, n (%)	7 (21.88)	7 (24.14)	0 (0.00)	0 (0.00)

**Table 3:** Clinical outcomes. Values are expressed as mean ± SD or percentages.

	Baseline (n = 30)	26 w (n = 28)	39 w (n = 27)	52 w (n = 25)	104 w (n = 20)
ODI, Mean ± SD	62.89 ± 19.24	35.73 ± 21.65	30.50 ± 17.87	31.35 ± 20.71	24.30 ± 18.99
*p*-value		<0.0001	<0.0001	<0.0001	<0.0001
SF-36, Mean ± SD	47.56 ± 25.42	54.60 ± 24.30	56.94 ± 23.21	52.64 ± 23.65	63.36 ± 22.13
*p*-value		0.1081	0.0428	0.2180	0.0070
VAS (back), Mean ± SD	53.10 ± 27.09	39.39 ± 23.87	25.30 ± 20.41	28.64 ± 23.15	19.45 ± 16.18
*p*-value		0.0462	<0.0001	<0.0001	<0.0001
VAS (leg), Mean ± SD	59.20 ± 33.12	31.93 ±28.80	26.37 ± 25.96	34.80 ± 29.63	20.95 ± 21.24
*p*-value		0.0015	0.0001	0.0061	<0.0001

## Data Availability

The data presented in this study are available on request from the corresponding author.
